# Cumulative Genetic Score of DRD2 Polymorphisms Is Associated with Impulsivity and Masked Semantic Priming

**DOI:** 10.1007/s12031-022-02019-5

**Published:** 2022-05-30

**Authors:** Simon Sanwald, Christian Montag, Markus Kiefer

**Affiliations:** 1 Department of Psychiatry and Psychotherapy III, Section for Cognitive Electrophysiology, Ulm University, Leimgrubenweg 12, 89075 Ulm, Germany; 2 Department of Molecular Psychology, Institute of Psychology and Education, Ulm University, Ulm, Germany

**Keywords:** Semantic priming, Dopamine, Impulsivity, Executive functions, DRD2

## Abstract

Individual differences in the magnitude of semantic priming effects are associated with executive functions (EF). Striatal dopamine has been shown to be associated with EF as well as impulsivity and could therefore be associated with differences in the magnitude of semantic priming. We investigated *n* = 155 individuals in an unmasked as well as in a masked semantic priming paradigm. We additionally assessed self-reported impulsivity and a cumulative genetic score (CGS) comprising six polymorphisms that have been found to be functionally relevant for the expression of the DRD2 gene. We found a significantly negative association between the DRD2 CGS and reaction time priming in the masked semantic priming paradigm. In addition, the DRD2 CGS was positively associated with self-reported impulsivity. Our findings complement previous research by showing a role of the DRD2 gene for masked semantic priming. Therefore, the investigation of genes within the dopamine system might improve our understanding of the genetic basis of impulsivity and semantic processing. Thus, the DRD2 CGS is of interest for clinical as well as experimental psychological research.

## Introduction

Access to the meaning of words is frequently investigated with the semantic priming paradigm (Meyer and Schvaneveldt 1971). In the semantic priming paradigm, a prime word is presented prior to a target word. The prime thereby facilitates the response to a semantically related target (Neely 1977, 1991): For instance, in a lexical decision task, in which real target words have to be discriminated from meaningless pseudowords, semantically related prime-target pairings yield shorter response latencies and a higher accuracy than semantically non-related prime-target pairs (semantic priming effect). Semantic priming effects depend either on controlled or on automatic semantic processing or a combination of both. Expectancy generation or semantic matching (a recognized semantic relation between prime and target indicates a word response in the lexical decision task) is regarded to depend on controlled semantic processing (Neely 1991). In particular, expectancy generation is assumed to operate only at stimulus onset asynchronies (SOAs) longer than 300 ms (Neely 1991), where semantic matching might also occur at earlier SOAs (Koivisto 1998).

In contrast, spreading activation (Collins and Loftus 1975) or preactivation of semantic features (Masson 1995) is considered to reflect automatic semantic processing, which can also occur for unconsciously perceived prime words. A masked priming paradigm can be used to investigate automatic semantic processing (Kiefer 2002; Marcel 1983): For instance, a random sequence of letters can serve as a mask, which is presented before and/or after the prime, thereby preventing its conscious perception (Breitmeyer and Öğmen 2006).

The magnitude of masked and unmasked priming effects, i.e. the difference in reaction times (RT) and error rates (ER) between semantically related and semantically non-related prime target pairings, varies considerably across individuals. It has been suggested that interindividual differences in priming at least partly depend on prefrontal functioning and are associated with executive function (EF) processes (Miyake et al. 2000; Posner and DiGirolamo 1998), i.e. the cognitive control of thought and action, given a common substrate for both aspects of cognition in prefrontal cortex (Kiefer et al. 2005). The prefrontal cortex is assumed to guide semantic retrieval (Thompson-Schill et al. 1999; Wagner et al. 2001) and serves to focus semantic access to close associates (Spitzer et al. 1993, 1994). Prefrontal dysfunction should thus result in an increase of semantic priming. Initial evidence in support of this hypothesis came from studies investigating thought-disordered patients with schizophrenia. In comparison to healthy controls, thought-disordered patients with schizophrenia, exhibiting prefrontal dysfunction, showed exaggerated conscious priming in particular for indirectly semantically related words (lemon-sweet) (Maher et al. 1996; Spitzer et al. 1994; Weisbrod et al. 1998) as well as exaggerated unconscious priming for directly semantically related words (tiger-stripes) (Kiefer et al. 2009). Furthermore, healthy participants with low EF (as measured with a digit span backward task) were shown to exhibit larger RT priming for indirectly associated prime-target pairings with visible primes (Kiefer et al. 2005).

Given this association between EF and semantic priming, genes impacting EF might also be able to explain variance in interindividual differences in semantic priming. There are to date only three studies investigating the molecular genetics of semantic priming examining polymorphisms of genes formerly associated with differences in EF (Berger et al. 2021; Reuter et al. 2009; Sanwald et al. 2020). One study found Met carriers of the brain-derived neurotrophic factor (BDNF) Val66Met polymorphism to show less RT and stronger ER priming in a masked semantic priming paradigm (Sanwald et al. 2020). When reanalyzing this data by means of drift diffusion models (Berger et al. 2021), drift rate and decision thresholds were lower in Met than in Val carriers suggesting a more superficial processing style in Met carriers. Finally, the catechol-o-methyl transferase (COMT) gene, coding for the postsynaptic enzyme that methylates released dopamine, has been suggested to be important for prefrontal functioning (Weinberger et al. 2001). The Val158Met polymorphism of the COMT gene has been associated with prefrontal functioning and EF in some studies (Barnett et al. 2008; Mier et al. 2010; Tunbridge et al. 2006), although other work and meta-analyses could not always substantiate this association (Barnett et al. 2008; Geller et al. 2017). Mirroring these inconsistent findings with regard to EF, COMT Val158Met was not significantly related to the magnitude of semantic priming (Reuter et al. 2009). While the COMT Val158Met polymorphism does not seem to play a role in semantic priming, other elements of the dopamine system might contribute. In the present study, we therefore examined the associations of single nucleotide polymorphisms (SNPs) in the DRD2 gene coding for the dopamine D2 receptor, executive functions and semantic priming.

Genetically determined variations of the D2 receptor, which is mainly found in the striatum (Jaber et al. 1996), are of interest, because functioning of the dopamine D2 receptor has frequently been associated with executive functions (Cervenka et al. 2008; MacDonald et al. 2009) and impulsivity (Buckholtz et al. 2010). Impulsivity can be defined as a predisposition promoting rapid, unplanned reactions to both internal or external stimuli without regarding the negative consequences of these reactions to oneself or others (Moeller et al. 2001). Striatal D2 receptor binding has been found to be positively associated not only with cognitive performance in tests of episodic memory but also with performance in non-episodic tasks depending on the examined location within the striatum (Cervenka et al. 2008).

With respect to semantic priming, previous studies postulated a major role of D1 as compared to D2/D3 receptors for controlled semantic processing in healthy individuals as well as in individuals suffering from Parkinson disease (Pederzolli et al. 2008; Roesch-Ely et al. 2006). Possibly, D2 receptor functioning is related to automatic semantic processing as elaborated below, although this issue has not yet been investigated. D2 receptor activity seems to have a key role in complex behaviors (Simpson et al. 2010). In addition, lower striatal D2 receptor availability has been consistently associated with impulsivity in previous studies (e.g. Lee et al. 2009). Accordingly, low levels of striatal dopamine have been associated with symptoms of attention deficit hyperactivity disorder (ADHD) (Moreno et al. 2010; Volkow et al. 2007). Conversely, patients suffering from schizophrenia show a hyperactive dopaminergic transmission at the D2 receptor (Abi-Dargham et al. 2000). This is intriguing since both disorders are associated with impulsivity and EF deficits (Toplak et al. 2005; Wing et al. 2013). In schizophrenia, deficits in EF and impulsivity are often attributed to abnormal firing of dopamine neurons in the striatum, which increases the salience of innocuous stimuli. This may increase noise in the system resulting in prefrontal dysfunction and thus negative symptoms and deficits in EF since inhibition of irrelevant stimuli does not function properly (Howes and Kapur 2009). Patients suffering from ADHD on the other hand have been shown to have deficits in reward anticipation due to reduced dopaminergic activity in the striatum (Tripp and Wickens 2009). Reward anticipation is important for learning because the dopamine response should transfer to cues that predict reinforcement in order to provide reinforcement at the cellular level when behavioral reinforcement is delayed (Tripp and Wickens 2009). Therefore, it has been postulated that an initial deficit in attention and reward processing (Fabio 2017; Tripp and Wickens 2009) is associated with a deficient automatization of simple tasks in patients suffering from ADHD. This in turn results in deficits in higher level cognition since cognitive load increases with deficits in automatic processing (Fabio 2017).

Accordingly, striatal dopaminergic neurotransmission has been postulated to play a key-role early in learning processes and automatically provides the prefrontal cortex with useful task specific representations (Villagrasa et al. 2018). Based on these findings (Fabio 2017; Tripp and Wickens 2009; Villagrasa et al. 2018), it is possible that lower dopaminergic neurotransmission at striatal D2 receptors decreases automatic semantic activation due to deficient reward-related learning processes contributing to the establishment of semantic associations. Consequently, individuals carrying variants of DRD2 polymorphisms associated with reduced striatal D2 activity might exhibit lower masked semantic priming effects. Unmasked priming, i.e. priming with visible primes might be less affected by DRD2 polymorphisms because controlled semantic processing, which is involved in visible priming, depends primarily on D1 receptor activity and to lesser extent on D2 receptor activity (Pederzolli et al. 2008; Roesch-Ely et al. 2006).

The human D2 receptor has two isoforms D2L and D2S generated by alternative splicing. These isoforms are expressed in the same cell types in a ratio that usually favors the expression of D2L. D2L differs from D2S by the insertion of 29 amino acids (Picetti et al. 1997). The gene coding for the D2 receptor (DRD2) is located on human chromosome 11q 22–23 (Jaber et al. 1996) and comprises several polymorphisms (Jönsson et al. 1999). The results of previous studies investigating the associations between single polymorphisms of the DRD2 gene and EF produced heterogeneous results (Klaus et al. 2019).

Instead of examining each polymorphism separately, associations between genotype and behavioral data can be analyzed by means of a cumulative genetic score (CGS) (Lancaster et al. 2016). The aggregation of the usually small effects of single polymorphisms with similar functionality may increase the variance explained while avoiding type I error inflation due to multiple testing (e.g. Enge et al. 2020). Therefore, in this study, we investigate six DRD2 polymorphisms that have been shown to be functionally relevant in previous studies: rs1800497, rs2283265, rs6277, rs12364283, rs2242592 and rs4648317. The minor T/A1 allele of the rs1800497 also known as Taq1A polymorphism is located ~ 10 kilobases downstream of the DRD2 gene in the ankyrin repeat and kinase domain containing 1 (ANKK1) gene and is associated with a reduced D2 receptor density and reduced D2 receptor binding (Jönsson et al. 1999; Ritchie and Noble 2003; Tunbridge et al. 2019). The minor T allele of the rs2283265 has been found to be associated with a decreased expression of the DRD2 short splice variant relative to the long variant and reduced performance in working memory and attentional control tasks (Zhang et al. 2007). The C allele of the rs6277 also known as C957T polymorphism was shown to be associated with a lower receptor affinity of D2 receptor (Hirvonen et al. 2009). The major T allele of the rs12364283 has been associated with decreased DRD2 expression (Zhang et al. 2007). Furthermore, carriers of the rs2242592 risk allele C exhibited lower D2L expression than major T allele carriers (Kaalund et al. 2014). Last, individuals carrying the minor T allele of the rs4648317 showed increased prolactin concentrations attributed to reduced D2 binding as compared to CC homozygotes (Fukui et al. 2011).

We investigated the interrelations of unmasked as well as masked semantic priming, EF and a DRD2 CGS in 155 individuals. We hypothesized that the more risk variants of the six DRD2 polymorphisms an individual carries, the more impaired the striatal dopamine system. Therefore, we expected reduced masked priming effects and higher self-reported impulsivity in these individuals. Again, and in detail: We assumed that a high DRD2 CGS is associated with high self-reported impulsivity based on previous studies in healthy individuals (Buckholtz et al. 2010; Lee et al. 2009) and the aforementioned association between low levels of striatal dopamine and symptoms of ADHD (Moreno et al. 2010; Volkow et al. 2007). In addition, we assumed that a high DRD2 CGS is associated with lower masked semantic priming effects due to the assumed role of striatal D2 for reward-based learning and automatic processing (Fabio 2017; Tripp and Wickens 2009; Villagrasa et al. 2018). Based on previous results (Kiefer et al. 2005), we expected that EF scores would be negatively related to priming. However, since both high and low activity of the striatal dopamine system can be associated with low EF as seen in studies investigating ADHD and schizophrenia (although the presumed underlying mechanisms differ) (Toplak et al. 2005; Wing et al. 2013), we did not assume a direct or linear association between DRD2 CGS and EF.

## Patients and Methods

### Participants

One-hundred eighty-eight native German speakers with normal or corrected-to-normal vision participated in the study. Data from this sample was analyzed in previous studies with regard to a BDNF polymorphism (Berger et al. 2021; Sanwald et al. 2020). Sixteen participants were later excluded because they reported a diagnosis of a neurological or psychiatric disorder. Furthermore, 5 participants showing extremely high mean reaction times across all trials of the priming paradigms (more than threefold interquartile range above the median), 1 participant without priming data due to a technical error, 1 participant who had a mean error rate of 48.13% across all trials of the masked priming paradigm (near the chance performance of 50% errors) and 4 participants who had an accuracy of more than 61.25% (confidence interval of chance performance) in the masked prime identification task (described below) were excluded from analyses. Moreover, six participants had to be excluded from analyses because genotype data for one or more of the DRD2 polymorphisms was missing. Data of the remaining *n* = 155 participants (*n* = 120 females, 77.4%; *n* = 138 right-handed, 89.0%; *n* = 16 left-handed, 10.3% and *n* = 1 ambidextrous, 0.6%), all healthy Caucasian volunteers recruited in Ulm, Germany, were included in the analyses. Mean age was *M* = 22.41 years (*SD* = 3.69 years, *range*: 18 to 49 years). All participants were students at Ulm University with the exception of two participants having lower levels of education. The study was approved by the local ethics committee at the University of Ulm. All participants gave written informed consent to participate in the study.

### Genotyping

After DNA was extracted from buccal cells, purification was conducted by means of the MagNA Pure 96 system using the MagNa Pure 96 DNA kit from Roche Diagnostics, Mannheim, Germany. Genotyping of the DRD2 polymorphisms was implemented on a MALDI-TOF platform (Agena; Massarray 4) by Varionostic, Ulm, Germany. In accordance with previous studies using CGS analysis (e.g. Enge et al. 2020), participants’ DRD2-CGS was calculated by adding the numbers of alleles previously associated with lower dopaminergic neurotransmission, as outlined above, assuming a linear model. In short, the number of T alleles of the rs1800497 (TT: 6, TC: 46, CC: 103; HWE: *χ*^2^(1) = 0.09, *p* = .762), the number of T alleles of the rs2283265 (TT: 4, TG: 40, GG: 111; HWE: *χ*^2^(1) = 0.03, *p* = .862), the number of C alleles of the rs6277 (TT: 45, TC: 78, CC: 32; HWE: *χ*^2^(1) = 0.03, *p* = .866), the number of T alleles of the rs12364283 (TT: 126, TC: 27, CC: 2; HWE: *χ*^2^(1) = 0.16, *p* = .688), the number of C alleles of the rs2242592 (TT: 76, TC: 66, CC: 13; HWE: *χ*^2^(1) = 0.06, *p* = .802) and the number of T alleles of the rs4648317 (TT: 3, TC: 37, CC: 115; HWE: *χ*^2^(1) = 0.00, *p* = .992) were added, resulting in a CGS ranging from 0 to 12 (Table 1).

**Table 1:** Frequencies of single CG scores

CGS	Number	Percent	Cumulative percent
1	1	0.60	0.60
2	34	21.90	22.60
3	10	6.50	29.00
4	45	29.00	58.10
5	30	19.40	77.40
6	16	10.30	87.70
7	15	9.70	97.40
8	4	2.60	100.00

### Assessment of Working Memory and Executive Functions

The capacity of verbal working memory as well as the ability to manipulate stored information (EF) was assessed using two digit span tasks adapted from the HAWIE-R (Tewes 1994), a digit span forward and backward task. Since we were interested in EF, the capacity to manipulate stored information, we only included the digit span backward task in our analyses. The digit span backward was defined as the maximum number of digits a participant was able to recall correctly in reverse order. Length of digit sequences ranged from two to eight. There were two sets of digits for each span (whereas digits differed between the two digit sequences of each length). The maximum digit span was the maximum number of digits the participant was able to recall correctly at least once. Mean number of digits recalled correctly was *M* = 4.26 digits (*SD* = 1.07 digits, *range*: 2 to 7 digits).

### Barrat Impulsiveness Scale (BIS-11)

The BIS-11 (Patton et al. 1995) assesses the behavioral construct of impulsiveness. It comprises 30 items describing impulsive or non-impulsive behaviors. Items are answered on a 4-point-scale from 1 (rarely/never) to 4 (almost always/always). For descriptive statistics, see Table 2. Reliability of the BIS-11 total was acceptable to high with *α* = 0.79.

**Table 2:** Descriptive statistics of the BIS-11 for the three second-order factors (Attentional Impulsiveness, Motor Impulsiveness, Nonplanning Impulsiveness) and the six first-order factors (Attention, Cognitive Instability, Motor, Perseverance, Self-Control, Cognitive Complexity)

	*n*	*Minimum*	*Maximum*	*M*	*SD*
BIS-11 total	155	43.00	83.00	61.63	8.56
Attentional Impulsiveness total	155	9.00	27.00	16.54	3.35
Attention	155	6.00	19.00	10.44	2.42
Cognitive Instability	155	3.00	10.00	6.10	1.53
Motor Impulsiveness total	155	15.00	32.00	21.59	3.31
Motor	155	7.00	22.00	13.63	2.62
Perseverance	155	4.00	13.00	7.96	1.49
Nonplanning Impulsiveness total	155	13.00	37.00	23.49	4.11
Self-Control	155	6.00	19.00	11.91	2.79
Cognitve Complexity	155	7.00	18.00	11.58	2.26

The total as well as second and first order factors are reported

### Procedure

After completing a German version of the “Edinburgh Handedness Inventory” (Oldfield 1971) identifying the hand used to respond, the main experiment started. We assessed semantic priming using a lexical decision task requiring the participant to decide whether the target was a real word or a pseudoword. Pseudowords were lexically meaningless pronounceable letter strings (e.g. “Nempen”). They served as distractors. Primes were always German words. The instruction given to the participants was to answer as quickly and accurately as possible. Responses were given by pressing one of two keys on a response box. A key press with the index finger indicated the “word” response, whereas the key pressed with the middle finger indicated the “pseudoword” response. Reaction times for pseudoword trials were not analyzed. All participants completed the masked priming paradigm before being presented with the unmasked priming paradigm. Subjects performed 24 training trials at the beginning of each priming experiment. The experiments were programmed and presented by means of the ERTS software (Experimental Run Time System, Berifsoft, Frankfurt, Germany).

**Fig. 1:**
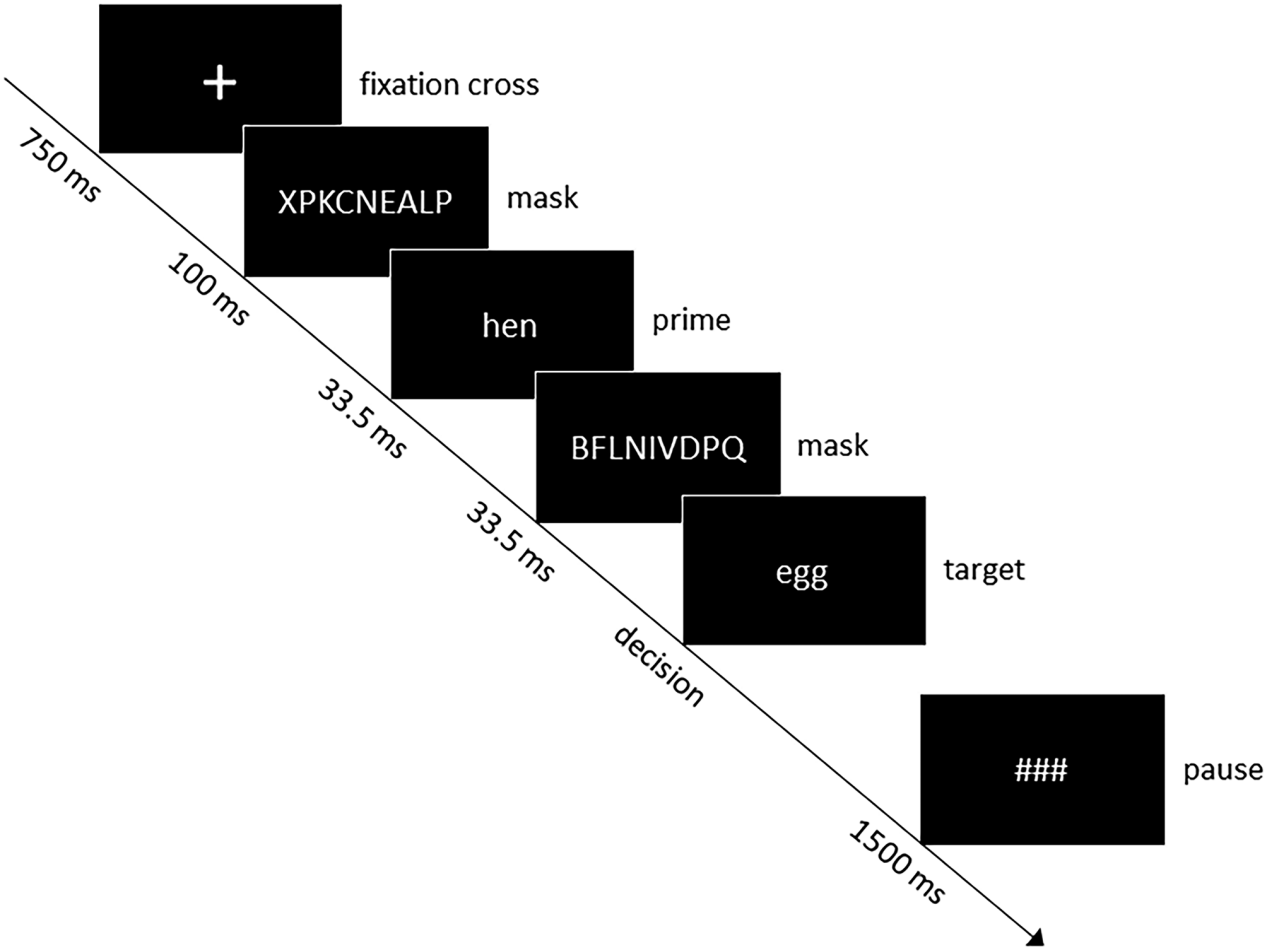
Schematic presentation of a single trial of the masked priming paradigm with a semantically related prime target pair

The masked priming paradigm consisted of 160 trials (80 word-word and 80 word-pseudoword pairs) and was adapted from previous experiments (Kiefer 2002; Kiefer and Brendel 2006; Reuter et al. 2009). While 40 of the word-word pairs were directly related (hen-egg), the other 40 word-word pairs were not related (leaf-car). Targets of the related conditions were matched for word length and word frequency (Kiefer 2002). All trials started with a fixation cross for 750 ms followed by a mask consisting of ten random letters shown for 100 ms. Thereafter, the prime was presented for 33.5 ms. Afterwards, a random letter mask was shown for 33.5 ms. Finally the target stimulus, a German word or a pseudoword, was presented until the participant’s decision. For a schematic representation of the masked priming paradigm, see Fig. 1. Participants were uninformed on the prime shown between the two masks.

**Fig. 2:**
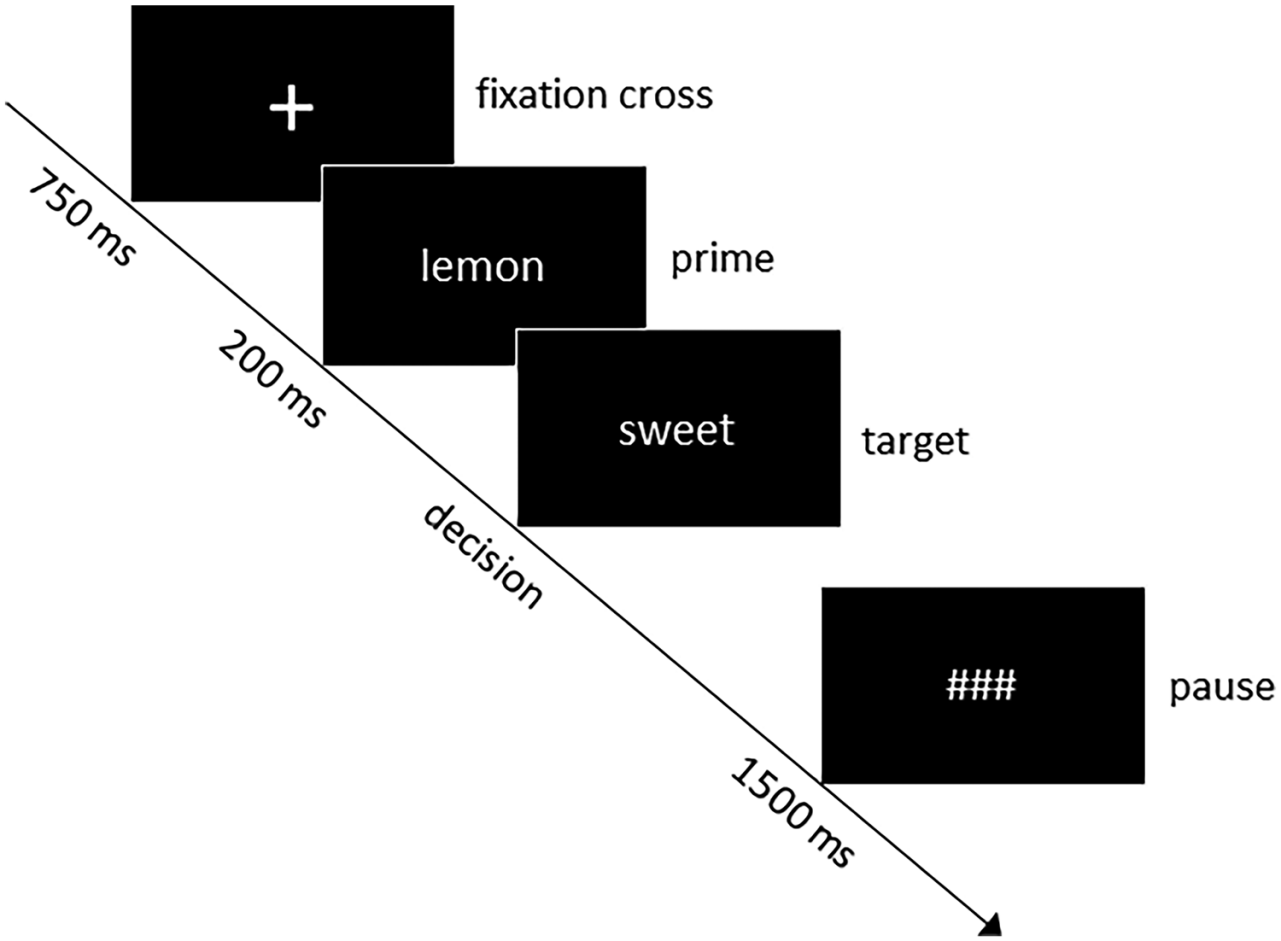
Schematic presentation of a single trial of the unmasked priming paradigm with an indirectly related prime target pair

The unmasked priming paradigm was adapted from Kiefer et al. (2005). It consisted of 108 trials; 54 word-word and 54 word-pseudoword pairs. The word-word pairs consisted of 18 directly (hen-egg), 18 indirectly (lemon-sweet) and 18 non associated (leaf-car) pairs. Targets of the different semantic relatedness conditions were matched for word length and word frequency as well. All trials started with a fixation cross for 700 ms followed by the prime presented for 200 ms. The target stimulus was shown immediately thereafter until an answer was given. For a schematic representation of the unmasked priming paradigm, see Fig. 2.

Regarding the masked as well as the unmasked priming paradigm, trials with RT ± 2 *SD* from an individual’s mean RT across all trials were excluded from analysis. In the masked priming paradigm, an average of 1.3% of all word-word trials were excluded. In the unmasked priming paradigm, an average of 1.1% of all word-word trials were excluded from further analyses.

After the experiments, participants were debriefed on the existence of a prime and of the mask in the masked priming experiment at the beginning of the session. The debriefing was followed by a recognition task in order to assess if the participants had been able to identify the prime in the masked condition. The participants were therefore presented with 80 trials of the masked priming paradigm, 40 word-word and 40 word-letter string pairs. The instruction was to decide as accurately as possible whether the prime was a real word or a letter string comprising the repetition of the same capital letter (i.e. KKKKKKK). In case participants were unable to identify the prime, they were instructed to guess. Prior to the actual recognition task, five training trials were presented to ensure the participants’ understanding of the task while preventing learning or frustration. For descriptive statistics of the masked and unmasked priming paradigm, see Table 3.

**Table 3:** Descriptive statistics of the unmasked and the masked priming paradigm

	*n*	*Minimum*	*Maximum*	*M*	*SD*
*Unmasked*					
RT in ms					
Related	155	417.16	792.29	541.29	76.52
Indirectly related	155	444.49	806.41	576.78	76.78
Non-related	155	442.51	893.09	589.48	82.04
ER in %					
Related	155	0.00	22.22	1.00	2.84
Indirectly related	155	0.00	27.78	2.76	4.49
Non-related	155	0.00	27.78	5.73	5.78
*Masked*					
RT in ms					
Related	155	458.45	720.04	565.37	57.11
Non-related	155	453.97	768.07	585.30	61.94
ER in %					
Related	155	0.00	22.50	1.94	2.93
Non-related	155	0.00	15.00	3.21	3.30

### Statistical Analyses

We conducted statistical analyses using IBM SPSS Statistics (version 24, IBM, USA) as well as R with the *psych* package (R Development Core Team 2008; Revelle 2018).

We calculated Pearson’s correlation coefficients to investigate the association between the DRD2 CGS, the BIS-11 and EF (note that Spearman’s correlation coefficients provided similar results). We performed four (two for masked and two for unmasked priming) repeated measures ANCOVAs with RT (of correct trials)/ER as dependent variable, relatedness between prime and target as within-subjects factor and DRD2 CGS, digit span backward scores as well as age as covariates. Sex was not further investigated due to the small number of males, who participated in the present project. In order to further elucidate significant interactions between semantic relatedness and EF or DRD2 CGS found in the repeated measures ANCOVAs, we calculated partial Spearman’s correlation coefficients between the difference in RTs and EF or DRD2 CGS controlling for age and the other respective covariate (EF or DRD2 CGS). Statistical significance was determined at *p* < 0.05; all tests were two-tailed.

## Results

### Masked Prime Identification Task

Mean accuracy in the masked prime identification task was 50.90% (*SD* = 5.51%) and significantly differed from the chance level of 50% (*t*(154) = 2.04, *p* = .043). Mean accuracy was not significantly associated with DRD2 CGS (*r* =  − 0.14, *p* = .158).

The difference between participants’ z-standardized hit rates (correct responses to words) and z-standardized false alarms (incorrect responses to pseudowords) was calculated in order to get *d’* sensitivity measures (Green and Swets 1966). Neither *d’* for all (*M* = 0.05, *SD* = 0.32, *t*(154) = 1.89, *p* = .06), nor *d’* for semantically related (*M* = 0.04, *SD* = 0.39, *t*(154) = 1.30, *p* = .19) or non-related trials (*M* = 0.05, *SD* = 0.38, *t*(154) = 1.91, *p* = .06) did significantly differ from zero.

### Correlation Analyses of DRD2 CGS, Impulsivity and Digit Span Backward

The DRD2 CGS was significantly associated with self-reported impulsivity across all BIS-11 scales with *r* = 0.23, *p* = .004. The DRD2 CGS was also significantly associated with two out of three second-order factors and half of the first-order factors comprising the BIS-11 (Table 4). There was no significant association between the DRD2 CGS and EF as measured by the digit span backward task (*r* = 0.02, *p* = .78).

**Table 4:** Pearson’s correlation coefficients and *p*-values of the associations between the DRD2 CGS and BIS-11 scales/subscales

BIS-11 scales and subscales		DRD2 CGS
Attentional Impulsiveness	*r*	.212**
	*p*	.008
Attention	*r*	.291**
	*p*	.000
Cognitive Instability	*r*	.004
	*p*	.959
Motor Impulsiveness	*r*	.229**
	*p*	.004
Motor	*r*	.208**
	*p*	.009
Perseverance	*r*	.144
	*p*	.073
Nonplanning Impulsiveness	*r*	.121
	*p*	.135
Self-Control	*r*	.225**
	*p*	.005
Cognitve Complexity	*r*	− .059
	*p*	.468

** *p* < .01

### Priming Effects in the Unmasked and Masked Priming Paradigm and DRD2CGS

The two repeated measures ANCOVAs with RT or ER as dependent variables revealed a significant priming effect for RTs but not for ER in the unmasked priming paradigm (Table 5). Participants showed shorter response latencies when prime and target were directly semantically related as compared to semantically indirectly and non-related prime-target pairings (direct semantic priming). Trials with indirectly related prime-target pairings yielded shorter RTs than trials, in which prime and target were not semantically related (indirect semantic priming).

**Table 5:** Priming effects for the masked and unmasked priming paradigm

Behavioral data	Related (A)	Indirectly related (B)	Non-related (C)	Repeated-measures ANCOVA	Post hoc (*Tukey HSD*)
Unmasked					
RT	*M* = 541.29	576.29	589.48	*F*(2,302) = 7.50	A < B < C
In ms	*SD* = 76.52	76.78	82.04	*p* = .001	
Covariates: age, DRD2 CGS, digit span				${\eta }_{\text{p}}^{2}$= .05	
ER	*M* = 1.00	2.76	5.73	*F*(1.68,253.70) = 2.06	
In %	*SD* = 2.86	4.49	5.78	*p* = .129	
Covariates: age, DRD2 CGS, digit span				${\eta }_{\text{p}}^{2}$= .01	
Masked					
RT	*M* = 565.37	-	585.30	*F*(1,151) = 12.25	A < C
In ms	*SD* = 57.11	-	61.94	*p* = .001	
Covariates: age, DRD2 CGS, digit span				${\eta }_{\text{p}}^{2}$= .08	
ER	*M* = 1.94	-	3.21	*F*(1,151) = .62	
In %	*SD* = 2.93	-	3.30	*p* = .433	
Covariates: age, DRD2 CGS, digit span				${\eta }_{\text{p}}^{2}$= .00	

*M* mean, *SD* standard deviation, ${\eta }_{\text{p}}^{2}$ partial eta squared, *RT* reaction time, *ER* error rate

We also found priming effects in the masked priming paradigm. There were significant priming effects for RTs but not for ER (Table 5). Participants showed significantly faster reactions in trials with semantically related primes and targets as compared to trials with non-related primes and targets.

In the unmasked priming paradigm, there were no other significant effects aside from the aforementioned effects considering RT priming.

**Fig. 3:**
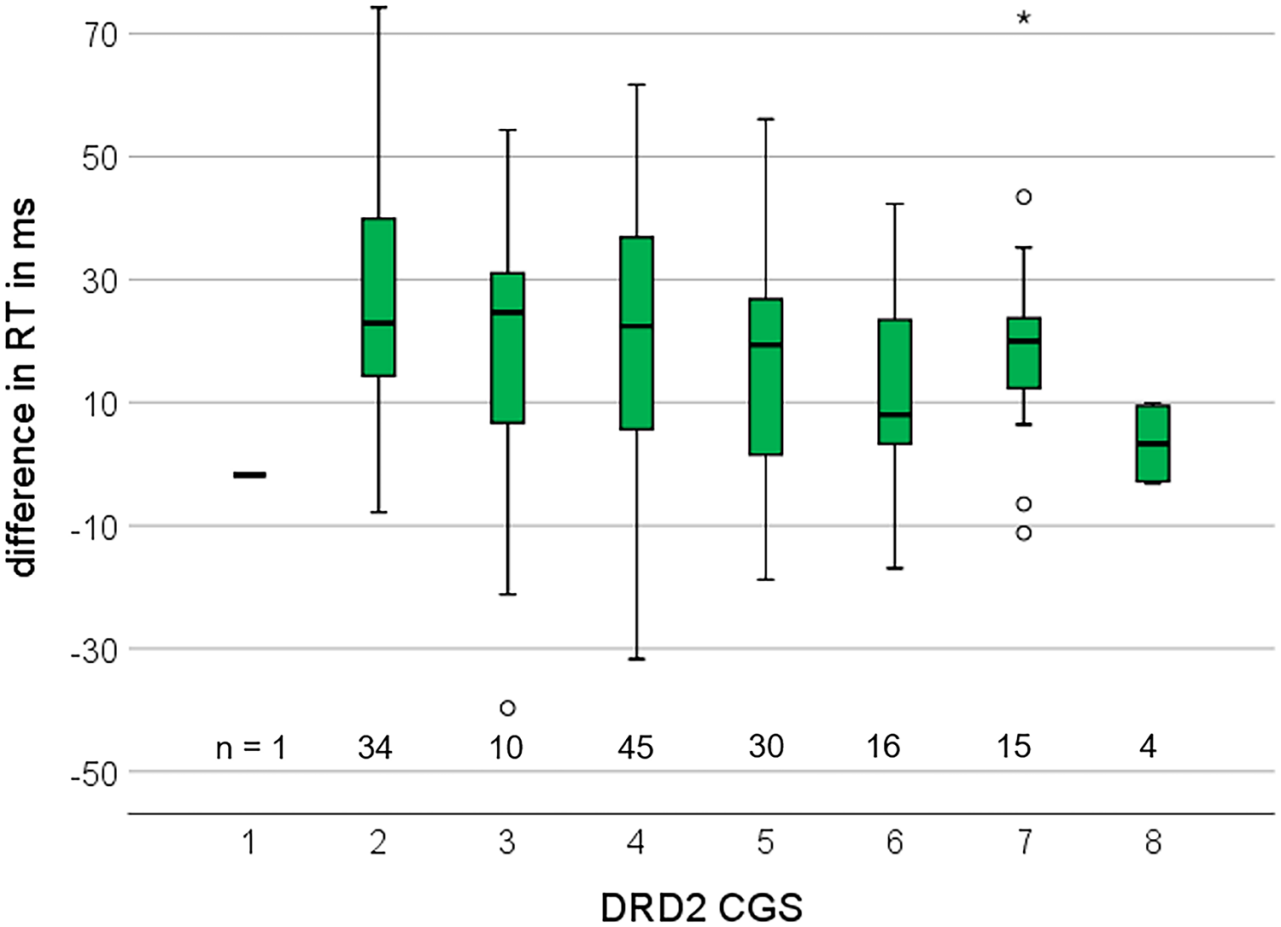
Boxplots of RT priming effects in the masked semantic priming paradigm as a function of DRD2 CGS

In the masked priming paradigm, there was a significant interaction between relatedness and DRD2 CGS (*F*(1,150) = 5.06, *p* = .026, *partial η*^*2*^ = .03). DRD2 CGS was significantly negatively associated with the difference between RTs of semantically related and semantically non-related prime target pairings when calculating partial Spearman’s correlation coefficients with age and digit span backward as covariates (*r*_*SP*_ =  − .20, *p* = .013; Fig. 3). Thus, participants with a high DRD2 CGS tended to show reduced RT priming in the masked priming paradigm. There were no other significant main effects or interactions for masked RT priming. For ER, there was a significant interaction between relatedness and digit span backward scores (*F*(1,150) = 7.29, *p* = .008, *partial η*^*2*^ = .05). Higher digit span backward scores were significantly positively associated with ER priming (difference in ER between trials with semantically related and semantically non-related prime target pairings) when calculating Spearman’s correlation coefficients with age and DRD2 CGS as covariates (*r*_*SP*_ = .20, *p* = .015). There were no other significant main effects or interactions for masked ER priming.

## Discussion

In this study, we hypothesized that the more risk variants of the six DRD2 polymorphisms an individual carries, the more impaired the striatal dopamine system. Based on the results of Buckholtz and colleagues (2010), we assumed that a high DRD2 CGS is associated with high self-reported impulsivity. Furthermore, we assumed that a high DRD2 CGS is associated with lower priming effects in the masked semantic priming paradigm.

In accordance with our hypothesis of a positive association between DRD2 CGS and trait impulsivity, we found significantly positive associations between overall impulsivity (and most of the scales of the BIS-11) and DRD2 CGS. These results are in line with previous studies showing an inverse relationship between D2 receptor availability and trait impulsivity (Buckholtz et al. 2010; Lee et al. 2009). Furthermore, the results are in line with prior evidence linking low levels of striatal dopamine with symptoms of ADHD (Moreno et al. 2010; Volkow et al. 2007). In addition, two meta-analyses report significant associations between DRD2 polymorphisms, impulsive behaviors and ADHD (Blum et al. 1995; Wu et al. 2012). The exact mechanism by which dopamine D2 receptors affect impulsivity are unclear. However, it has been postulated that the fronto-striatal dopamine system plays an important role for inhibition processes (Lee et al. 2009). Our study extends previous research by providing evidence that a CGS with respect to DRD2 polymorphisms may represent a marker for impulsivity. The dopamine D2 receptor seems to be closely linked with impulsivity, even on the level of genetics.

We did not find a significant association between DRD2 CGS and EF. This is, however, not surprising in light of prior studies describing a complex relationship between D2 receptors and EF with D2 receptor stimulation either increasing or decreasing working memory performance and different response profiles in dopamine neurons depending on their anatomical location and projection target (for a review, see Ott and Nieder 2019). Our results are also in accordance with a recent meta-analysis which did not find a significant association between polymorphisms of the DRD2 gene and EF (Klaus et al. 2019).

We found the expected priming effects regarding RTs, i.e. faster RTs in trials with semantically related prime-target pairings as compared to trials with indirectly or non-related primes and targets (Neely 1977, 1991). This was true for the unmasked as well as the masked priming paradigm. However, even though we found the expected pattern of lower ERs in trials with semantically related primes and targets as compared to trials with indirectly or non-related prime-target pairs on a descriptive level, this effect did not reach significance in the repeated measures ANCOVA. A possible explanation is that inclusion of the covariates could have masked small ER priming effects.

There was a negative association between DRD2 CGS and RT priming in the masked priming paradigm. As expected, individuals with a high number of risk alleles showed reduced RT priming. Our results suggest that reduced striatal D2 receptor availability is associated with reduced automatic semantic processing. This aligns with our assumption that the influence of D2 receptors on learning processes and on automatically provided task-specific representations (Villagrasa et al. 2018) results in deficits in the establishment and automatic activation of semantic networks. There are studies postulating a major role of D1 as compared to D2/D3 receptors for controlled semantic processing in healthy individuals as well as individuals suffering from Parkinson Disease (Pederzolli et al. 2008; Roesch-Ely et al. 2006). Whereas the role of prefrontal D1 receptors for working memory and executive functions in general is well established (Durstewitz and Seamans 2002), more recent theories suggest prefrontal as well as striatal involvement in complex behaviors and attribute a key role to striatal D2 receptors (Simpson et al. 2010). Thus, the findings of our study complement earlier studies on dopamine and semantic priming. Building upon theories of cortico-striatal circuitry dysfunctions in schizophrenia (Simpson et al. 2010), we suggest that D1 as well as D2 receptors are involved in semantic processing. It is possible that D1 receptors are an important factor for controlled semantic processing while automatic semantic processing is associated with D2 receptor availability. This aligns with results from studies showing exaggerated priming in patients suffering from schizophrenia (Kiefer et al. 2009; Maher et al. 1996; Spitzer et al. 1994; Weisbrod et al. 1998) who exhibit a hyperactive dopaminergic transmission at the D2 receptor (Abi-Dargham et al. 2000). Our findings are also relevant for research on semantic processing in ADHD: Patients suffering from ADHD show reduced dopaminergic neurotransmission in the striatum (Moreno et al. 2010; Volkow et al. 2007). One could therefore hypothesize that patients with ADHD show reduced priming effects in a masked semantic priming paradigm. Patients with ADHD could have less coherent semantic networks due to deficits in D2 receptor-associated reward learning and automatization (Fabio 2017; Tripp and Wickens 2009; Villagrasa et al. 2018) resulting in less semantic priming. First indications in favor of this hypothesis come from a study showing decreased semantic priming in children with reading disability, a condition that is often comorbid with ADHD (Betjemann and Keenan 2008).

We found no genotype effect regarding unmasked semantic priming. This is in line with previous studies on BDNF Val66Met and semantic priming which used similar behavioral data (Berger et al. 2021; Sanwald et al. 2020). It is possible that visible primes induce the application of strategic priming mechanisms masking the small effects of genotype on semantic priming (Sanwald et al. 2020). Another explanation could be that D2 receptor availability affects only automatic but not controlled semantic processing as outlined above.

Moreover, there was a significant association of EF and ERs in the masked semantic priming paradigm. High EF was significantly positively associated with ER priming. While this finding seems to contradict earlier studies reporting a negative association between EF and unmasked semantic priming (Kiefer et al. 2005), our results are difficult to interpret since including EF in the ANCOVA resulted in non-significant ER priming. Additionally, we used a masked semantic priming paradigm while Kiefer and colleagues (2005) used an unmasked semantic priming paradigm with different SOAs. Furthermore, associations between EF and semantic priming have been described as complex even interacting with the type of semantic relatedness between prime and target (Heyman et al. 2015). In line with our results, reduced semantic priming has been reported in individuals with mild cognitive impairment (Brambati et al. 2012). In addition, previous studies report that attention to the prime is a prerequisite for masked priming and that masked priming effects can be amplified when attentional resources are available (Kiefer and Brendel, 2006; Martens and Kiefer 2009; Naccache et al. 2002). At first glance, this contradicts findings of exaggerated priming effects in thought-disordered patients with schizophrenia (Kiefer et al. 2009; Maher et al. 1996; Spitzer et al. 1994; Weisbrod et al. 1998). However, in patients with schizophrenia, exaggerated priming effects are hypothesized to be a result of a lack of inhibition leading to disinhibited spreading of activation in semantic networks (Kiefer et al. 2009; Maher et al. 1996; Spitzer et al. 1994; Weisbrod et al. 1998). We did not investigate inhibition but rather the manipulation of working memory contents. Therefore, the association between EF and masked semantic priming should be investigated assessing EF in detail using tests for working memory, updating, shifting and inhibition as well as different prime-target relationships.

Last, it is worth mentioning that mean accuracy in the masked prime identification task differed significantly from chance level. However, we examined a relatively large sample for an experimental study and mean accuracy is only slightly above 50%. Therefore, it is possible that this difference only became significant due to our sample size. Additionally, *d’* sensitivity measures did not significantly differ from zero indicating that the prime was not discriminable from the letter string.

Some limitations need to be taken into account interpreting the results of the present study. First, even though we had a large sample for an experimental study, the investigation of the genetic basis of semantic priming might need larger samples, something which is hard to achieve for experimentally working psychologists (Montag et al. 2020; Montag and Reuter, 2014). Second, we measured EF by means of a digit span backward task. In future studies, the investigation of EF using additional tasks, for instance a Stroop or flanker task (Eriksen and Eriksen 1974; Stroop 1935), will be of major importance to unravel the complex relationship between different domains of EF and semantic priming. Furthermore, even though we investigated six DRD2 polymorphisms, even more polymorphisms as well as other genes of the dopamine system and other systems interacting with the brain dopamine system need to be investigated to achieve a comprehensive understanding of the genetic basis of semantic processing. Last, given the well-known problems in replicating findings in genetic association studies in related research areas (Border et al. 2019), it is of high relevance to see what other groups will observe when replicating the present work.

In conclusion, we provide novel results of associations between a DRD2 CGS and self-reported impulsivity as well as masked semantic priming. The DRD2 CGS we investigated is therefore of interest for clinical and experimental psychological research. Our results suggest that the DRD2 CGS could be a genetic marker for highly impulsive individuals. Furthermore, striatal D2 receptors and not only D1 receptors might be important for semantic processing. This study is therefore a starting point for future research on the associations between genetic variants and classical experimental psychological experiments.

## Data Availability

The datasets generated during and/or analyzed during the current study are available from the corresponding author on reasonable request.
